# The relationship between parent-child attachment and fertility intention: the mediating role of family resilience and the moderating role of gender moderating

**DOI:** 10.3389/fpsyg.2025.1669639

**Published:** 2025-10-06

**Authors:** Gui-Ying Li, Zhi-Qi You, Guang-Hui Yang

**Affiliations:** ^1^Center for Mental Health Education for College Students, Henan Polytechnic, Zhengzhou, Henan, China; ^2^Department of Social Work, Huazhong Agricultural University, Wuhan, Hubei, China; ^3^School of Education, Anyang Normal University, Anyang, Henan, China

**Keywords:** parent-child attachment, family resilience, fertility intention, father attachment, mother attachment, family beliefs, family strength, gender

## Abstract

**Objective:**

The study aims to explore how parent-child attachment relates to college students fertility intention, mediated by family resilience and moderated by gender .

**Methods:**

A study surveyed 2186 college students using various scales to assess parent-child attachment, family resilience, fertility intention. Data analysis included descriptive stats, partial correlations, mediation and moderation effects.

**Results:**

(1) Parent-child attachment (including its sub-dimensions: father attachment and mother attachment), family resilience (including its sub-dimensions: family beliefs and family strength), and fertility intention show significant positive correlations; (2) Total scores of parent- child attachment and family resilience positively predict fertility intention– Specifically, father attachment (rather than mother attachment) and family beliefs (rather than family strength) positively predict fertility intention; (3) After controlling for variables such as family economic status, family beliefs partially mediate the relationship between father attachment and future fertility intention; (4) Both segments of the mediating pathway are significantly moderated by gender– Male’s father attachment has a stronger promoting effect on family beliefs than female’s, male’s family beliefs directly drive fertility intention, while female’s family beliefs do not directly influence fertility intention.

**Conclusion:**

This study clarified the core role path of ‘father attachment → family beliefs → fertility intentions,’ and found that men are more likely to form family beliefs through father attachment, which can directly drive their fertility intentions, while women’s fertility intentions need to be improved by alleviating actual fertility costs. This result not only provides new evidence for the theoretical mechanism of ‘ parent-child relationships affecting fertility intentions,’ but also offers practical basis for formulating fertility intention enhancement strategies differentiated by gender and dimension.

## 1 Introduction

In the current international context of population aging and declining birth rates, how to enhance fertility intentions has become a focal topic among researchers. University graduates are expected to become the main force in future fertility, and their fertility intentions will significantly influence future population trends. Therefore, studying college students’ future fertility intentions holds great significance. Existing research primarily explores the impact of macro factors such as policy, economy, and education on fertility decisions (Cao et al., 2025, China). Although some international studies have examined factors influencing fertility intentions from a micro-family perspective–such as South Korean research finding that parental role confidence and family support environment predict young people’s fertility intentions (Lee and Lee, 2025; Kim and Yi, 2024), Japanese studies demonstrating that improved parent-child interaction quality and family function can enhance children’s parenting competence and psychological burden (Uji and Kawaguchi, 2024), and European research revealing harmonious family relationships effectively boost fertility intentions (Mönkediek and Bras, 2018)–there remains a scarcity of studies exploring direct correlations between parent-child relationships and college students’ future fertility intentions from a micro-family system perspective both domestically and internationally. Family system theory posits that households serve as crucial conduits for transmitting fertility concepts and values, with family members’ internal dynamics being closely linked to reproductive intentions (Cox and Paley, 2003, USA). As fertility costs-to-benefits ratios continue to decline and workplace gender segregation in household division intensifies, strategically leveraging families as platforms to cultivate healthy marital and reproductive attitudes among young adults could significantly impact future population trends. Therefore, understanding how family relationships and functions influence fertility preferences holds both theoretical significance and practical relevance for population development.

## 2 Literature review and hypotheses

### 2.1 Parent-child attachment and fertility intention

Parent-child attachment refers to the profound emotional bond formed between an individual and their parents during early childhood, providing a “secure base” for exploring the world (Bowlby, 1982, United Kingdom). Research conducted in China indicates that the internal working model developed through positive parent-child attachment relationships can enhance positive cognition, emotional stability, stress management, and interpersonal adaptability, significantly impacting physical and mental health development (Yin et al., 2021, China). Consequently, strong parent-child attachments may positively influence reproductive intentions. First, according to the inter-generational transmission mechanism model of attachment (Amato and Cheadle, 2005, USA), such bonds foster positive emotional connections toward future offspring, thereby increasing reproductive intention. Research shows that the social interaction between parents and children leads to similarities in many beliefs and behaviors of both sides, including fertility intentions and fertility behaviors (Di Nallo and Oesch, 2023). Studies in other countries have also reached similar conclusions. A study in South Korea found that childhood emotional abuse is also a predictor of not wanting to have children (Lee et al., 2021), while a study in China discovered that childhood traumatic experiences negatively predict fertility intentions (Qian and Huang, 2024), a Finnish study revealed that early family environment is associated with the ideal number of children (Karhunen et al., 2023). Second, based on the interpersonal self-expansion theory (Aron and Aron, 1986, USA), individuals within supportive parent-child relationships can boost self-efficacy by acquiring resources, adopting positive perspectives, and strengthening self-identity, leading to more proactive attitudes toward child-rearing. Additionally, healthy parent-child attachments help cultivate positive personality traits like optimism, patience, and perseverance–these qualities reduce the possibility of parenting anxiety caused by “difficult parenting” discourse, enhancing parenting self-efficacy and thus fertility intention (Zhang et al., 2024, China; Ma et al., 2024, China).

From a theoretical perspective, maternal and father attachment may exert distinct influences on individuals’ fertility intentions. According to the Independent Organization Hypothesis of Multiple Attachment Theory (Bowlby, 1982, United Kingdom; Schaeffer and Emerson, 1964, United Kingdom), children form multiple attachment relationships with different caregivers (e.g., fathers, mothers, friends, teachers) through unique interaction patterns. These varied attachment relationships impact psychological development differently rather than being determined by a single attachment pattern. Mothers maintain an innate closeness with their children, typically serving as primary “safety bases” during early childhood, emphasizing daily care and emotional communication. This helps cultivate empathy and caregiving skills in children, which indirectly shapes their fertility attitudes. In contrast, fathers primarily assume roles as “exploratory guides” providing economic support, life planning, and social value shaping–factors that more directly influence children’s decisions regarding marriage and reproduction (Xu and Feng, 2025, China; Zou et al., 2020; Rodriguez Ruiz et al., 2019, Spain; Kumar and Mattanah, 2016, USA). Cross-generational transmission studies (Amato and Cheadle, 2005) indicate that father attachment has a more significant predictive role in family decisions (including fertility) due to patriarchal norms within collectivist cultures. Therefore, it is necessary to analyze mother attachment and father attachment separately to explore their different effects on college students’ fertility intention.

Based on the above theoretical differences, this study puts forward hypothesis 1: (1) Parent-child attachment positively predicts fertility intention; (2) The direct prediction effect of father attachment on fertility intention is stronger than that of mother attachment.

### 2.2 The mediating role of family resilience

Family resilience serves as a crucial link between parent-child attachment and individual fertility intentions. Family resilience refers to a family’s adaptive capacity and recovery ability when facing stress and challenges (Walsh, 1996, USA). This resilience is influenced by factors such as socioeconomic status, parental education level, family structure, family functioning, and parent-child relationships. Notably, the positive impact of parent-child attachment on family resilience has been validated by relevant theories and research. Attachment theory posits that healthy parent-child relationships form the cornerstone for families to function positively (Bowlby, 1982, United Kingdom), not only promoting the physical and mental well-being of family members but also enhancing their ability to withstand potential risks and foster resilient development. Empirical studies conducted in China further demonstrate that strong parent-child attachment helps improve family functioning, strengthen resilience, and enhance psychological adaptation (Guo, 2023, China).

According to observational learning theory (Bandura, 2015, USA), children develop coping strategies for future parenting pressures through observing and imitating their parents’ stress management. Research indicates that family resilience correlates positively with children’s social support, psychological resilience, self-efficacy, and hope levels (Song et al., 2023, China). A resilient family environment subtly cultivates children’s stress resistance, problem-solving skills, and social adaptability (Walsh, 1996, USA; Liu et al., 2024, China), enabling them to better shoulder marital and parenting responsibilities. Household resilience determines the extent to which families can maintain their ability to raise children in the face of economic/social shocks, and differences at this level affect young people’s confidence in starting a family and having children (Bawati et al., 2025). The “social disruption” hypothesis suggests that the disruption of family relationships may reduce fertility support, increase fertility pressure and thus lower fertility (Lan, 2021). A study from China shows that family resilience can buffer the psychological stress of pregnancy (Kang et al., 2022). Studies also demonstrate that parent-child attachment influences social adaptation by affecting family functioning (Zeng et al., 2020, China).

Based on this, we propose a research hypothesis 2–family resilience mediates the relationship between parent-child attachment and future fertility intention.

### 2.3 The moderating role of gender

The impact of family resilience on fertility intentions may vary by gender. From a cultural perspective, influenced by China’s patriarchal family traditions (Shi and Zheng, 2023, China), men hold dominant positions in families as economic pillars and representatives of authority. Men and their families possess greater decision-making power and discourse rights regarding fertility. Moreover, after marriage, more wives choose to live with their husbands’ families. The family relationships, economic support, and household environment (i.e., family resilience) of men profoundly influence their offspring’s fertility decisions. Therefore, the family resilience of women’s original families has little impact on their future fertility intentions. Additionally, influenced by China’s traditional gender role division of “men handle external affairs while women manage internal matters,” since men serve as primary economic pillars, they tend to participate less in childcare and household chores. These responsibilities naturally fall to women, allowing men to focus more on career development and personal self-actualization while women cannot prioritize professional growth (Eagly and Wood, 2016). When deciding whether to have children, women must weigh multiple factors: conflicts between childbirth and work, contradictions between household duties and self-actualization, tensions in mother-in-law and daughter-in-law relationships, and concerns about physical changes (Shi and Zheng, 2023, China; Qian and Huang, 2024, China). From a psychological perspective, Bandura’s (1997) self-efficacy theory (USA) indicates that men’s sense of family superiority and satisfaction with career value realization shape their “parental self-efficacy,” enhancing their confidence in assuming the father’s role. In contrast, women’s sense of subordination in the in-laws’ household and psychological stress from childbirth-work conflicts significantly diminish their “parental self-efficacy,” weakening their confidence in fulfilling maternal roles. Previous studies have also shown that women’s fertility intentions are significantly lower than men’s (Shi and Zheng, 2023, China), as female fertility intentions are constrained by various complex factors beyond family resilience. Based on this, this study proposes Hypothesis 3a: Gender moderates the relationship between family resilience and fertility intention–Specifically, family resilience has a stronger positive predictive effect on male fertility intention than females, indicating the second stage of the gender-regulated mediating pathway (“parent-child attachment → family resilience → fertility intention”).

Parent-child attachment may have gender differences in its impact on family resilience. Influenced by China’s traditional “son preference” concept, males typically assume primary family responsibilities and hold a higher status within the family. According to Bandura’s self-efficacy theory, a higher family status enables male parent-child attachment to enhance family resilience, manifested through stronger family harmony, support, and stress resistance capabilities. Existing studies often include gender as a moderating variable of family function, and its moderating effect has been widely supported empirically (Li et al., 2024, China). Based on the above arguments, this study proposes the hypothesis 3b: gender will moderate the first part of the mediating path of “parent-child attachment → family resilience → future fertility intentions.”

## 3 Research methods

### 3.1 Subjects

Using the cluster convenience sampling method, we conducted online surveys among college students from three universities in Henan Province, with class-level participation. A total of 2,355 questionnaires were collected. After excluding invalid responses such as those with excessively long or short completion times and repetitive answers, 2,186 valid responses were obtained, achieving a 92.82% effective response rate. Participants had an average age of 17–22 years (mean 19.52 ± 1.32), male 1041 (47.6%), female 1145 (52.4%); only child 160 (7.3%), non-only child 2026 (92.7%); rural students 1749 (80.0%), urban students 261 (11.9%), urban students 176 (8.1%).

### 3.2 Research tools

#### 3.2.1 Parent-child attachment scale

The Parent-Child Attachment Scale used in this study is the Chinese-adapted version of the Inventory of Parent and Peer Attachment (IPPA). It was originally developed by Arms-den and Green-berg (1987), adapted for Chinese samples by Wang (2007), China, and revised by Raja. The simplified version contains two sub-scales: Father Attachment and Mother Attachment, each consisting of 10 items across three dimensions: communication, trust, and detachment. The detachment dimension employs a reverse-scored 5-point scale (1 = completely inconsistent to 5 = completely consistent). Higher scores indicate greater secure attachment levels.

In this study, the Cronbach’s α for the sub-scales of father-child and mother-child attachment was 0.86 and 0.84, respectively, while the total scale showed a Cronbach’s α of 0.91. To further validate the scale validity, confirmatory factor analysis (CFA) was conducted on both sub-scales. Although the initial fit was poor, moderate theoretical and statistical adjustments resulted in good model fit: χ^2^/df = 4.35, RMSEA = 0.042, CFI = 0.90, and TLI = 0.90, indicating strong structural validity. For discriminant validity, the average variance extraction values (AVE) for father-child and mother-child attachment sub-scales were 0.58 and 0.56, both exceeding 0.5. The square roots of AVE (0.76 and 0.74) also surpassed the correlation coefficients between the two sub-scales (0.68), the correlation with family beliefs (0.56), father-child attachment with family strength (0.63), mother-child attachment with family beliefs (0.58), and mother-child attachment with family strength (0.62), demonstrating excellent discriminant validity.

#### 3.2.2 Family resilience

The Family Resilience Scale used in this study (Dai, 2008, China) includes two core sub-dimensions: (1) Family Belief (three factors: interpretation of difficulties, positive outlook, and life excellence)–reflecting the family’s cognitive orientation toward challenges; (2) Family Strengths (seven factors: problem-solving, intimate harmony, social support, organizational order, emotional sharing, clear communication, and collaborative coordination)–reflecting the family’s behavioral ability to cope with stress. Theoretically, Family Belief (especially “positive outlook”) helps individuals maintain optimism about future childrearing challenges, while Family Strengths (especially “problem-solving” and “social support”) enhances the ability to cope with practical pressures (e.g., childcare resource shortages)–both directly promoting fertility intention. The scale uses a 5-point Likert scale (1 = not applicable to 5 = applicable), with Question 30 being an inverse scoring item. Higher scores indicate greater family resilience.

In this study, the Cronbach’s α for the sub-dimensions of family beliefs and family strength was 0.92 and 0.96, respectively, while the total scale showed a Cronbach’s α of 0.97. Confirmatory factor analysis (CFA) was conducted to validate the construct validity of these sub-dimensions. Although the initial model exhibited poor fit, moderate theoretical and statistical adjustments resulted in a well-fitted model with χ^2^/df = 3.58, RMSEA = 0.057, CFI = 0.90, and TLI = 0.91, indicating strong construct validity. For discriminant validity, the average variance (AVE) values for family beliefs and family strength dimensions were 0.85 and 0.83, both exceeding 0.5. The square roots of AVE (0.92 and 0.91) surpassed the correlation coefficients between the two dimensions (0.90), father-child attachment (0.56), father-family attachment (0.63), mother-child attachment (0.58), and mother-family attachment (0.62) with family beliefs, demonstrating excellent discriminant validity.

#### 3.2.3 Fertility intention

Referring to previous studies (Qian and Huang, 2024), this study measured the future fertility intention of college students by the sum of three indicators: fertility possibility, ideal number of children and ideal age of childbirth.

#### 3.2.4 Control variables

Research indicates that fertility intentions are influenced by gender and family socioeconomic status (Cao et al., 2025; Qian and Huang, 2024). Therefore, this study incorporates gender and family socioeconomic status (SES) as control variables. Following the approach of Qu and Zou (2013), we measured family socioeconomic status (SES) using a composite index comprising four indicators: fathers occupation, mothers occupation, fathers education level, and mothers education level. Higher scores indicate greater family socioeconomic status. Additionally, age, being an only child, and place of origin are also included as control variables.

### 3.3 Statistical analysis

SPSS 26.0 was used for statistical analysis, and PROCESS 3.5 were used for mediation effect and moderated mediation effect analysis.

## 4 Results

### 4.1 Common method bias

To prevent common method bias (Zhou and Long, 2004, China), procedural controls (anonymous responses, reverse phrasing) were implemented. Harmon’s single-factor analysis showed 10 factors with eigenvalues >1, and the first factor explained 35.34% of the variance (less than 40%), indicating minimal common method bias.

### 4.2 Correlation analysis

The analysis results (see Table 1) reveal significant correlations among the core variables in this study. Significant positive correlations exist between fertility intention and parent-child attachment, father-child attachment, mother-child attachment, family resilience, family beliefs, and family strength. Specifically: The correlation coefficient between parent-child attachment and fertility intention was 0.188 (*p* < 0.001, 95% *CI* [2.106, 2.256], Cohen’s *d* = 2.181), the correlation coefficient between father attachment and fertility intention was 0.206 (*p* < 0.001, 95% *CI* [2.132, 2.282], Cohen’s *d* = 2.207), the correlation coefficient between mother attachment and fertility intention was 0.136 (*p* < 0.001, 95% *CI* [2.049, 2.197], Cohen’s *d* = 2.123); the correlation coefficient between family resilience and fertility intention was 0.176 (*p* < 0.001, 95% *CI* [2.045, 2.193], Cohen’s *d* = 2.119); the correlation coefficient between family belief and fertility intention was 0.160 (*p* < 0.001, 95% *CI* [2.019, 2.167], Cohen’s *d* = 2.093); and the correlation coefficient between family strength and fertility intention was 0.178 (*p* < 0.001, 95% *CI* [2.055, 2.203], Cohen’s *d* = 2.129). These correlations were not only statistically significant (*p* < 0.001), but also had a 95% *CI* excluding 0 and Cohen’s *d*-values > 0.8, indicating that these correlations were small but of practical significance (Cohen, 1988).

**TABLE 1:** Correlation analysis of research variables (n = 2186).

Predictor	*M*	*SD*	1	2	3	4	5	6
1. Parent-child attachment	3.791	0.612	–					
2. Father attachment	3.710	0.696	0.924***	–				
3. Mother attachment	3.871	0.639	0.909***	0.680***	–			
4. Family resilience	3.895	0.597	0.678***	0.622***	0.621***	–		
5. Family belief	3.937	0.599	0.620***	0.560***	0.577***	0.956***	–	
6. Family strengths	3.872	0.617	0.685***	0.633***	0.622***	0.988***	0.900***	–
7. Fertility intention	7.317	2.204	0.188***	0.206***	0.136***	0.176***	0.160***	0.178***

***p < 0.001, and so on.

### 4.3 Multivariate linear regression analysis

To further clarify the independent impacts of core variables, their sub-dimensions, and control variables on fertility intention, we conducted two multivariate linear regression analyses using fertility intention as the dependent variable.

In the first analysis, independent variables included Parent-Child Attachment (Total Score) and Family Resilience (Total Score), with control variables being age, being an only child, place of origin, gender, and family socioeconomic status. The results showed that Parent-Child Attachment (β = 0.158, *t* = 5.996, *p* < 0.001), Family Resilience (β = 0.090, *t* = 3.405, *p* < 0.001), and Gender (β = 0.754, *t* = 19.448, *p* < 0.001) were significant positive predictors of fertility intention (Gender as a dummy variable: male = 1, female = 0; same below). Family socioeconomic status (SES) (β = −0.072, *t* = −3.514, *p* < 0.001) was a significant negative predictor. However, age (β = 0.012, *t* = 0.645, *p* = 0.519), being an only child (β = 0.030, *t* = 1.503, *p* = 0.133), and place of origin (β = −0.033, *t* = −1.595, *p* = 0.111) showed no significant predictive effects.

In the second multiple regression analysis, independent variables included sub-dimensions of both parent-child attachment and family resilience–father attachment, mother attachment, family beliefs, and family strength. Control variables remained age, being an only child, place of origin, gender, and family socioeconomic status. Results indicated that father attachment (β = 0.141, *t* = 4.984, *p* < 0.001), family beliefs (β = 0.127, *t* = 2.834, *p* < 0.01), and gender (β = 0.756, *t* = 19.222, *p* < 0.001) were significant positive predictors of fertility intention. Family socioeconomic status (β = −0.071, *t* = −3.490, *p* < 0.001) was a significant negative predictor. Mother attachment (β = 0.038, *t* = 1.345, *p* = 0.179) and family strength (β = −0.036, *t* = −0.749, *p* = 0.454) showed no significant predictive effects. Age (β = 0.012, *t* = 0.644, *p* = 0.520), being an only child (β = 0.031, *t* = 1.548, *p* = 0.122), and place of origin (β = −0.034, *t* = −1.646, *p* = 0.100) also lacked significant predictive significance.

### 4.4 Mediation analysis: father attachment → family belief → fertility intention

Based on the aforementioned multiple regression results, the subsequent mediation analysis will focus on testing the core pathway of “father attachment → family belief → fertility intention,” rather than the generalized “parent-child attachment total score → family resilience total score → fertility intention.” This is because the dimensions of “mother attachment” and “family strength” have been proven to lack significant predictive power, and their inclusion in the model may lead to biased mediation effect testing. Additionally, control variables are limited to gender and family socioeconomic status as two significant factors. Using SPSS macro program PROCESS, Model 4 is employed to examine the mediating effect of family belief between father attachment and fertility intention, with significance verified through a bias-corrected percentile Bootstrap method.

**FIGURE 1:**
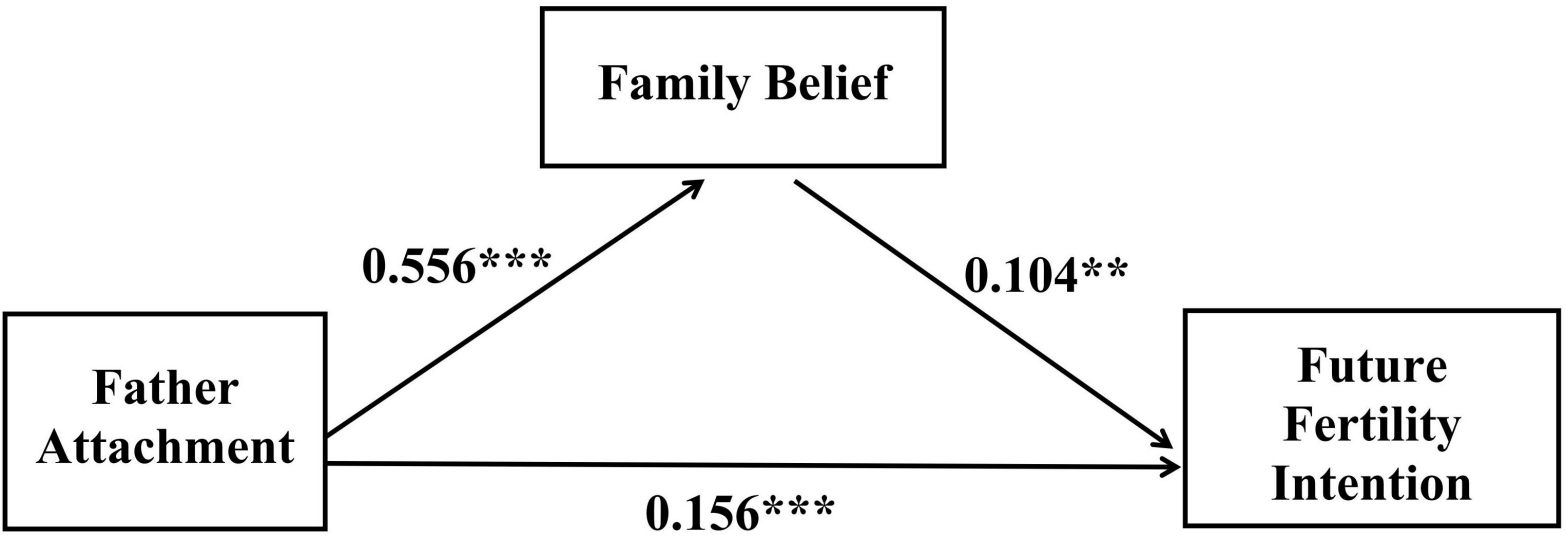
The mediating effect of family beliefs between father attachment and fertility intentions. **p < 0.01, ***p < 0.001.

The results indicate (see Figure 1 and Table 2) that after controlling for gender and family economic status, father attachment significantly positively predicts college students’ fertility intention (β = 0.214, *t* = 11.040, *p* < 0.001), with a significant regression equation (R^2^ = 0.183, *F* = 162.945, *p* < 0.001). Father attachment also significantly predicts family beliefs (β = 0.556, *t* = 31.550, *p* < 0.001, 95% *CI* [0.517, 0.597]), with a significant regression equation (R^2^ = 0.323, *F* = 346.791, *p* < 0.001). When introducing the mediating variable (family beliefs), family beliefs still significantly predict fertility intention (β = 0.104, *t* = 4.459, *p* < 0.001, 95% *CI* [0.055, 0.154]). While father attachment (β = 0.156, *t* = 6.693, *p* < 0.001, 95% *CI* [0.111, 0.202]) remains significant, its direct prediction coefficient for fertility intention decreases from 0.214 to 0.156. Bootstrap analysis reveals a significant mediating effect of family beliefs (*b* = 0.058, 95% *CI* [0.030, 0.087]). The total effect of the model was 0.214, and the direct effect was 0.156. The mediating effect accounted for 27.10% of the total effect, and the direct effect accounted for 72.90% of the total effect. It can be seen that the partial mediating effect of family belief between father attachment and college students’ fertility intention was significant.

**TABLE 2:** Father attachment and fertility intention: test of the mediating effect of family beliefs.

Predictor	Total effect equation: fertility intention		Intermediate variable equation: family beliefs		Dependent variable equation: fertility intention	
	β	*t*	β	*t*	β	*t*
Father attachment	0.156	6.693***	0.556	31.550***	0.214	11.040***
Gender	0.750	19.388***	−0.111	−3.137**	0.738	19.050***
Socioeconomic status	−0.078	−4.015***	0.081	4.620***	−0.069	−3.578***
Family belief	0.104	4.459***				
Indicators R	0.436		0.568		0.428	
R^2^	0.190		0.323		0.183	
F	128.236***		346.791***		162.945***	

All variables are standardized. Gender is a dummy variable, male = 1, female = 0.

**p < 0.01,

***p < 0.001.

### 4.5 Moderated mediation analysis: the role of gender

We conducted moderation analysis using SPSS’s PROCESS Model 58. First, examining the first stage of gender-moderation mediation (father attachment → family beliefs), the results (see Table 3) showed that the interaction between father attachment and gender significantly predicted fertility intention (β = 0.092, *t* = 2.606, *p* < 0.01, 95% *CI* [0.013, 0.169]). Second, for the second stage of mediation, the interaction between family beliefs and gender demonstrated significant predictive effects on fertility intention (β = 0.148, *t* = 3.836, *p* < 0.001, 95% *CI* [0.063, 0.232]), indicating that gender moderates both the first and second stages of the mediation pathway.

**TABLE 3:** Testing of a moderated mediation model.

Predictor	Intermediate variable equation: family beliefs		Dependent variable equation: fertility intention	
	β	*t*	β	*t*
Father attachment × gender	0.092	2.606***		
Family belief × gender			0.148	3.836***
Father attachment	0.515	21.783***	0.160	6.871***
Family belief			0.026	0.821
Gender	−0.110	−3.135**	0.749	19.448***
Socioeconomic status	0.081	4.599***	−0.078	−4.056***
Fit index R	0.570		0.443	
R^2^	0.325		0.196	
F	262.481***		106.176***	

**p < 0.01,

***p < 0.001.

**FIGURE 2:**
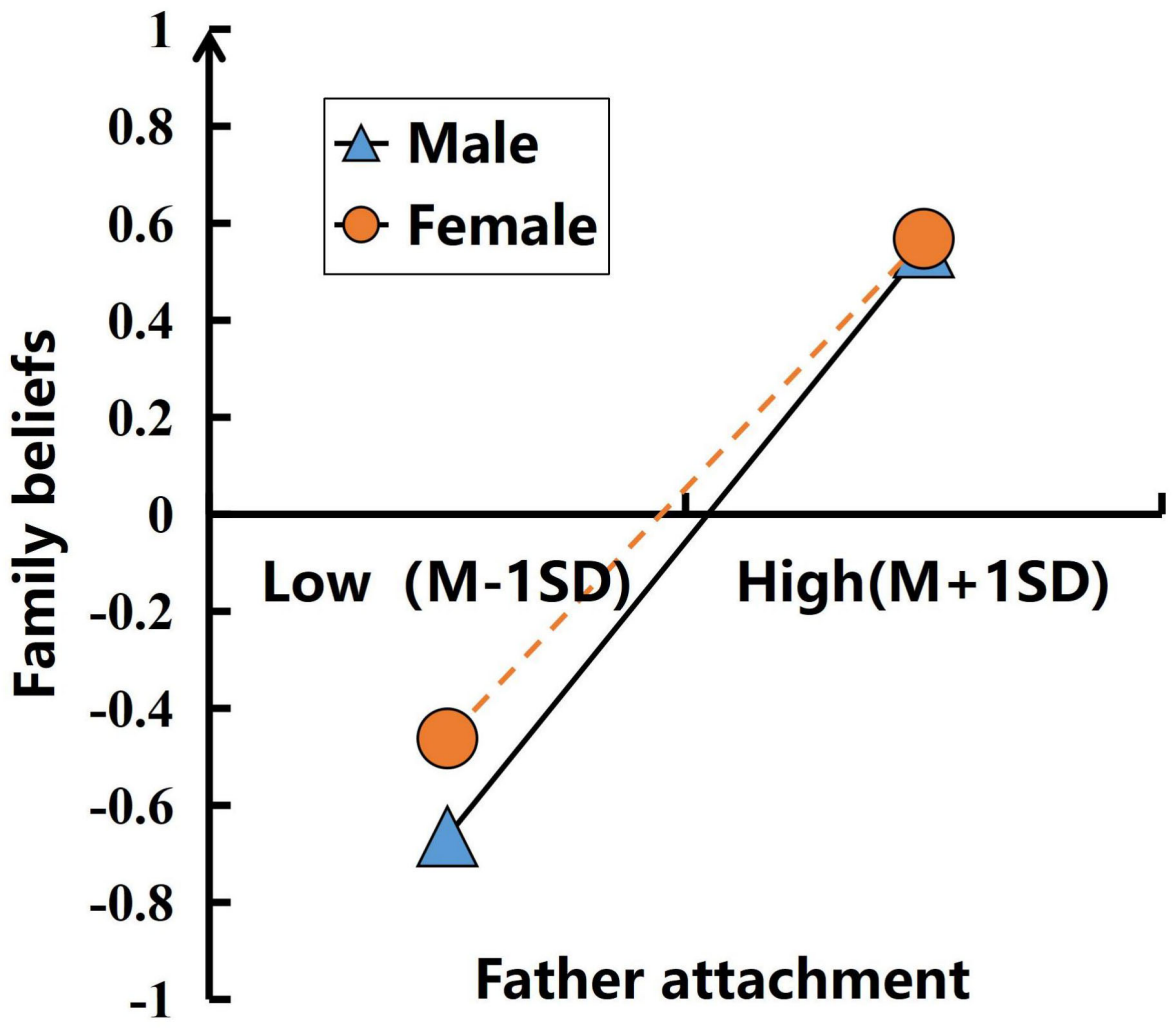
The moderating role of gender: father attachment → family beliefs.

**FIGURE 3:**
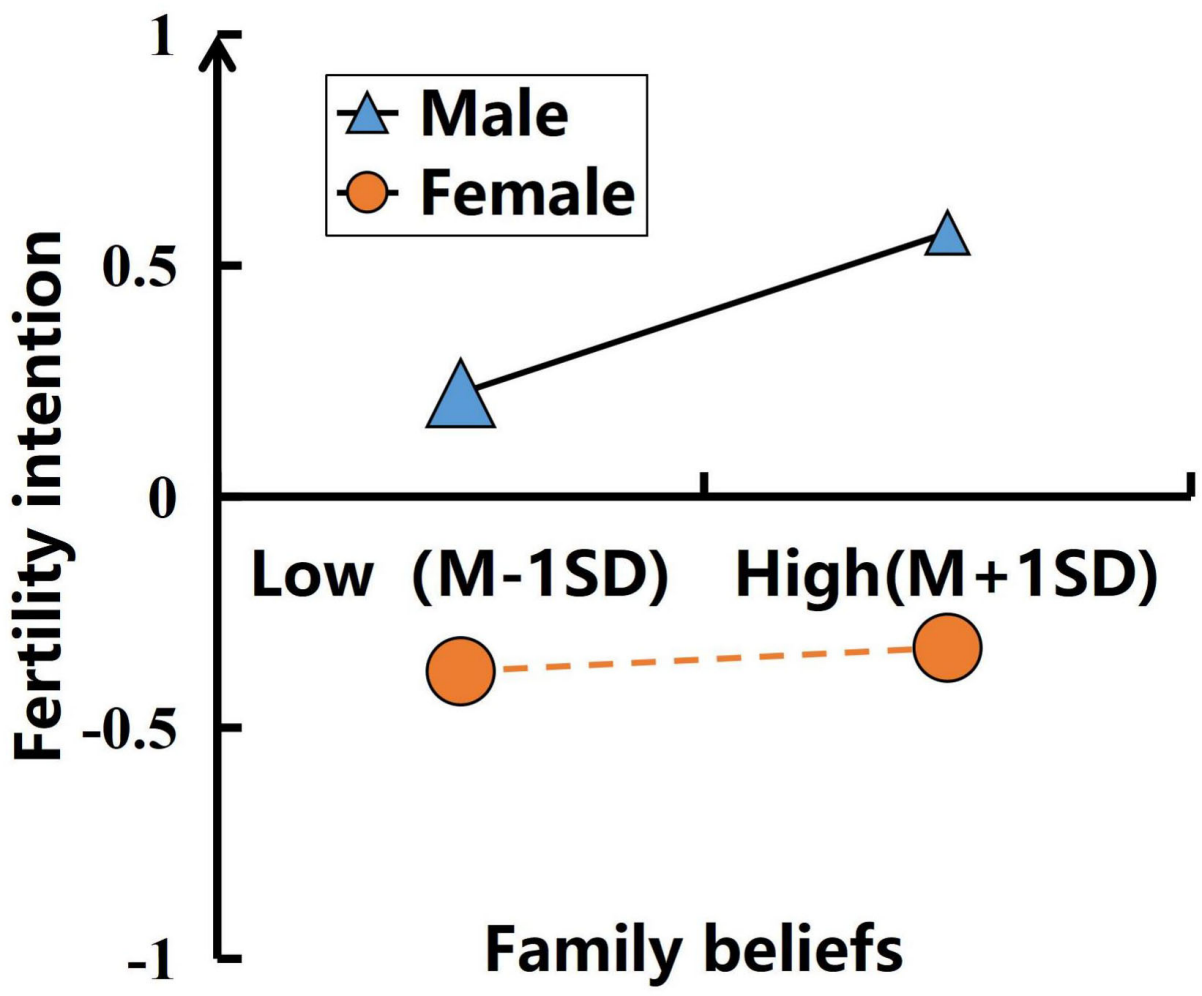
The moderating role of gender: family beliefs → fertility intentions.

To further explore this effect, we performed simple slope tests separately for male and female groups across the first and second mediation paths. In the first mediation path, we further divided the groups into low father attachment (*M*-1SD) and high father attachment (*M* + 1SD) subgroups for detailed analysis. The results showed (see Figure 2) that when the participants were male, the effect of father attachment on family beliefs was significant (*b* = 0.607, *t* = 23.041, *p* < 0.001); when the participants were female, the effect of father attachment on family beliefs was also significant (*b* = 0.515, *t* = 21.783, *p* < 0.001), but the predictive effect of male was significantly higher than that of female. In the second pathway of the mediation analysis, male and female participants were divided into low family belief group (*M*-1SD) and high family belief group (*M* + 1SD) for simple slope testing. The results (see Figure 3) showed that when participants were male, family belief significantly predicted fertility intention (β = 0.173, *t* = 5.884, *p* < 0.001); when participants were female, the effect was not statistically significant (β = 0.026, *t* = 0.821, *p* = 0.412).

## 5 Discussion

### 5.1 Multivariate regression analysis: from “total score effect” to “dimensional specificity” -a critical screening framework

The multivariate regression analysis at the total score level revealed that both Parent-Child Attachment Total Score and Family Resilience Total Score significantly predicted fertility intention. This suggests that from an “overall perception” perspective, individuals with higher parental attachment quality and stronger family resilience to stress are more likely to have their fertility intention positively stimulated. Additionally, among control variables, gender (positive correlation) and family socioeconomic status (negative correlation) significantly predicted fertility intention.

However, dimension-level multiple regression analysis indicates that only specific dimensions show significant predictive power, suggesting that not all dimensions impact fertility intentions.

First, the dimensions of parent-child attachment are differentiated. Unexpected research reveals that mother attachment shows no direct impact on fertility intentions (β = 0.038, *t* = 1.345, *p* = 0.179), while father attachment demonstrates significant predictive power (β = 0.141, *t* = 4.984, *p* < 0.001). This finding challenges the conventional assumption of “parental role equivalence in attachment dynamics.” The explanation stems from three core dimensions: differential functional roles, parental role expectations, and their correlation with reproductive decisions. According to attachment theory (Bowlby, 1982; Schaeffer and Emerson, 1964), parents fundamentally differ in their functions within children’s attachment systems, which directly affects their connection to “fertility intentions.” In both traditional and modern family structures, fathers are often perceived by children as the “family’s breadwinner” and “primary bearer of household responsibilities” (Shi and Zheng, 2023; Kumar and Mattanah, 2016; Amato and Cheadle, 2005). Children (both male and female) who directly observe or experience their fathers’ fulfillment of family responsibilities–such as household commitment, support for partners, and care for children–recognize that “the father’s role is effective, an indispensable part of the family, and can be fulfilled through effort.” This recognition subtly shapes their expectations about “forming families and assuming parental roles in the future,” making the correlation between father attachment and fertility intentions more direct and significant. In contrast, mother attachment to children primarily focuses on “daily emotional support” (e.g., emotional comfort and daily care). Even if children acknowledge the value of maternal roles, this doesn’t necessarily lead them to directly express “I want to become a parent and have children” –because fertility intentions also involve practical considerations like “child-rearing costs, time commitments, career conflicts, and partner support,” which mother attachment’s “emotional nourishment” struggles to address. Therefore, mother attachment cannot directly predict fertility intentions. However, it’s important to note that this doesn’t mean “mother attachment is unimportant” –rather, “its connection to fertility intentions requires more indirect pathways.” Subsequent statistical analysis revealed that mother attachment significantly predicts family beliefs (β = 0.573, *t* = 32.669, *p* < 0.001, *F* = 224.001), and family beliefs significantly predict fertility intentions (β = 0.127, *t* = 2.834, *p* < 0.01).This indicates that although mother attachment affects family beliefs, this influence is not transmitted to the level of fertility intention. Therefore, future research could explore the mechanism of mother attachment influencing fertility intentions through the pathway “mother attachment → other mediating variables (e.g., marital security) → fertility intention.” This phenomenon may also reflect the prevalent “father absence” in contemporary family education. While mothers naturally provide emotional support and care to children, fathers are often absent from most family structures. The absence of fathers (i.e., low-quality father attachment) makes children more aware of incomplete family functioning (such as parents failing to share responsibilities, mothers bearing sole burdens, and insufficient family support), which may lead to concerns about their own future family formation and childbearing. Consequently, the widespread presence of father absence renders father attachment a crucial predictor of fertility intentions, whereas mother attachment proves inadequate as a core indicator distinguishing levels of fertility willingness.

Secondly, the dimensions of family resilience showed differentiation. Only “family beliefs” were statistically significant, while “family strength” remained insignificant. This finding challenges the conventional assumption of uniform effects across all dimensions of family resilience. “Family beliefs” refer to internal value consensus within the family (positive interpretation of adversity and optimistic expectations for the future), whereas “family strength” pertains to practical coping resources (such as problem-solving capabilities, economic support, social assistance, and human capital). The results suggest that in family resilience, “family beliefs” serve as the core driver of fertility intentions, while the role of “family strength” remains inconclusive–Individuals’ decision to have children appears more dependent on their belief in maintaining a positive outlook and sharing burdens, rather than mere resource availability (possibly due to the independent explanatory effect of “economic status” in control variables, which may dilute the resource effect through multicollinearity). This discrepancy might stem from the fact that most participants in the sample have not yet faced real-life family challenges following childbirth, meaning their “capacity-level” needs remain unactivated, thereby masking the predictive power of fertility intentions.

Thirdly, control variables remained consistent across the two regressions. Both in aggregate scores and dimension analysis, gender (positive) and family socioeconomic status (negative) significantly predict fertility intention. This finding aligns with existing research consensus–For instance, women’s fertility intentions are often influenced by dual factors of traditional family values and perceived child-rearing costs, leading to more pronounced tendencies. While economic status remains the core practical consideration in fertility decisions, individuals with higher socioeconomic status may adopt more cautious attitudes due to career development pressures and rising child-rearing standards (Cao et al., 2025; Balbo et al., 2013). Among existing factors affecting fertility intentions, economic level has garnered the most attention from researchers. Studies (Wang and Shi, 2022, China) reveal that when income reaches a certain threshold, parents’ fertility intentions may decrease and their number of children diminishes–a phenomenon termed “high-income family fertility anxiety.” According to the “cost-benefit theory of childbearing” (Becker, 1960) and the “mutual substitution theory of child quality and quantity” (Becker and Lewis, 1973), high-income parents prioritize child quality, which increases financial burdens, time commitments, and energy costs in raising children. This sacrifices career development opportunities, thereby reducing fertility intentions (Yakita, 2020). Especially for women, as child-rearing requires more time from mothers, the cost of childbirth is higher for high-income mothers, which seriously reduces women’s willingness to have children (Park and Na, 2020, Korea).

### 5.2 Mediation analysis: partial mediating path from “father’s attachment → family belief → fertility intention”

Established the mediation analysis revealed that family belief partially mediates the relationship between father’s attachment and fertility intention (mediation effect accounting for 27.10%). This indicates that the influence of father’s attachment on fertility intention operates through two pathways: directly (e.g., when fathers’ fertility attitudes are transmitted to individuals) and indirectly–Specifically, stronger father’s attachment facilitates the formation of family belief in “effective coping with adversity and secure future livelihood,” thereby enhancing fertility intention.

Although the mediation effect accounts for a relatively small proportion of 27.10%, the establishment of “partial mediation” still holds theoretical significance: it demonstrates that family beliefs serve as a key “bridge” through which father attachment influences fertility intentions. First, the 27.10% mediation effect magnitude indicates that nearly one-third of the positive impact of father attachment on fertility intentions is transmitted through family beliefs. Even with a small effect size, it may still influence fertility rates at the population level when expanded to larger sample groups. Considering China’s massive youth population base (over 100 million young adults of childbearing age), even a modest increase in fertility intentions driven by family beliefs could translate into tens of thousands of potential additional births. Therefore, family beliefs act as a “buffer” against fertility pressures (such as economic stress and childcare burdens), and even this minor buffering effect can increase people’s likelihood of choosing to have children. Moreover, from an intervention perspective, family beliefs are more adjustable than father attachment (which forms during early childhood). Thus, interventions should focus on strengthening family beliefs (e.g., guiding families to cultivate positive parenting attitudes) to amplify the positive impact of father attachment on fertility intentions. Second, since fertility intentions are influenced by multiple complex factors (such as policies, economy, individual marital views, and individualistic tendencies), small effect sizes (20%–30% mediation effect) are common, indicating that this bridging role requires further explanation by incorporating other unaccounted variables (such as individual marital views and perceptions of fertility policies)– For instance, unmarried individuals’ fertility intentions may be more influenced by marital planning rather than family beliefs.

### 5.3 Moderation analysis: gender’s “two-stage moderating” effect on the mediating pathway

Gender showed significant moderating effects in both “father attachment → family belief” (stage 1) and “family belief → childbearing intention” (Stage 2), and the moderating patterns were significantly different between genders.

In the first stage of mediation, father attachment of both boys and girls significantly predicted family beliefs in a positive way, but father attachment of boys had a stronger promoting effect on fertility intention than that of girls. This may stem from differences in gender role socialization– Traditional perceptions portray men as dominant figures in family and society, representing authority and strength. As primary family leaders, decision-makers, and responsible parties (Amato and Cheadle, 2005; Xu and Feng, 2025, China), men are better equipped to handle pressures and challenges. Consequently, sons (men) tend to learn “family responsibilities and future planning” from their fathers, with paternal emotional support more readily translating into confidence in “family coping abilities.” In contrast, women’s construction of family beliefs is more influenced by maternal, peer, or romantic partner roles, making the impact of father attachment relatively weaker. This phenomenon may reflect the current lack of paternal involvement in family education.

In the second stage of mediation, family beliefs were a significant predictor of fertility intention only in the male group, but not in the female group. From a cultural perspective, influenced by the traditional Chinese patriarchal family system (Shi and Zheng, 2023, China), men are seen as the economic pillars and authoritative figures of the family. After marriage, more women choose to live with their husband’s family, so men’s perceived and actual family beliefs (interpretation of difficulties and positive outlook) more deeply influence the fertility decisions of the giver, while the family beliefs of women’s original families have little impact on their future fertility intentions. Additionally, influenced by the traditional Chinese gender role division of “men work outside, women work inside,” since men are the main economic providers, they participate less in childcare and household chores, which naturally fall to women. This allows men to focus more on career development and personal self-realization, while women cannot focus on their careers and face conflicts between work and childbirth (Shi and Zheng, 2023, China). For women, there exists an invisible cost of fertility penalties, such as a decline in wage income, fewer promotion opportunities, and even difficulty returning to the labor market (Kleven et al., 2019, Denmark). The Gender Revolution Theory posits that when men increase their involvement in household affairs, fertility levels will rise (Raybould and Sear, 2021). International research also indicates that career planning is the main reason for delaying childbirth among women, a finding replicated in more recent studies in Sweden (Gustafsson, 2005), the U.K. (Kneale and Joshi, 2008), Ireland (O’Donoghue et al., 2011), the U.S. (Amuedo-Dorantes and Kimmel, 2005; Miller, 2011) and Italy (Rondinelli et al., 2010). In short, compared with men, women have to bear additional specific costs of childbirth such as physiological risks, career interruption and childcare energy allocation. Only “positive beliefs” are not enough to translate into fertility intention, resulting in the failure of the predictive effect of family beliefs.

Finally, gender has a significant moderating effect on both the pre and post mediation paths, which is also consistent with the results of multivariate regression analysis–Gender is the strongest positive predictor of fertility intention (β = 0.754, *t* = 19.448, *p* < 0.001).

### 5.4 Research significance and enlightenment

This study investigates the impact of parent-child attachment on college students’ future fertility intentions while controlling for family socioeconomic status. Unlike previous macro-level analyses (such as policy, economic, and educational factors), this research reveals the connection between parent-child relationships and fertility intentions through a micro-level family system perspective, offering significant theoretical and practical implications.

The study theoretically enriched the research on the “parent-child relationship-birth intention” mechanism. The findings revealed that father attachment significantly predicts fertility intentions, while mother attachment shows no significant predictive power, laying the groundwork for future exploration of whether mother attachment influences fertility intentions through other mediating variables. The “dual-stage gender moderation effect” deepened our understanding of how gender mediates fertility intention, reinforcing gender’s pivotal role in this dynamic process.

Research also provides practical guidance for enhancing fertility intentions. First, strengthening paternal involvement is crucial to reinforce family beliefs. As families serve as key conduits for transmitting fertility concepts and values, and given that father attachment significantly predicts fertility intentions, male’s stronger influence on transforming “father attachment → family beliefs” –and male family beliefs directly drive fertility decisions, community and school programs like parenting workshops, fatherhood seminars, and experience-sharing sessions could boost fathers’ emotional engagement in children’s development, thereby encouraging childbearing. Second, policies should alleviate women’s anxiety about childbirth costs. While family beliefs alone cannot predict fertility intentions, the core issue lies in “beliefs failing to offset practical costs.” To boost women’s fertility willingness, efforts must focus on reducing actual expenses: improving childcare services, increasing maternity benefits, extending paternity leave (to ease women’s childcare burdens), and advancing workplace anti-discrimination policies (to ease career interruption anxiety). This creates a tangible foundation for translating “positive family beliefs” into actual fertility decisions. Thirdly, we must address the negative impact of socioeconomic status to avoid a “high-cost trap.” Negative correlations between family socioeconomic status suggest that high-income households may face “child-rearing anxiety” due to “excessively high upbringing standards” (e.g., prioritizing quality education over career advancement). To mitigate this, policy interventions like universal childcare services, balanced educational resource distribution, and flexible employment options for high-income parents could reduce their childcare cost expectations and ease their cautious approach to fertility decisions.

### 5.5 Research limitations and future research directions

Firstly, cross-sectional design cannot confirm the causal order between variables. For example, although we assume parent-child attachment → family resilience → fertility intention, it is also possible that individuals with higher fertility intentions may actively maintain closer parent-child attachment relationships; or family resilience may enhance the relationship between parent-child attachment and fertility intention. This uncertainty of bidirectional or reverse causality weakens the causal inference power of the mediation model and regression results–such as the mediation effect in this study being only 27.10%. Future research can adopt a three-wave longitudinal design: the first wave (freshman year of university) measures parent-child attachment, the second wave (senior year of university) measures family resilience, and the third wave (3 years after graduation) measures fertility intention–this design can more accurately verify the causal order between variables. Additionally, experimental interventions (such as implementing a parent-child attachment improvement plan or family resilience training for randomly sampled participants) can be conducted to actively verify whether changing these variables affects fertility intention, thereby strengthening causal inference.

Second, this study relied solely on self-report questionnaires, which may be influenced by social desirability bias–For example, participants might overestimate positive parent-child attachment or underestimate low fertility intentions to conform to societal expectations. Future research could supplement objective measurement indicators: (1) behavioral intention tasks (e.g., requiring participants to choose between parenting-related activities and career-related activities, using indicators of participation in premarital/career education programs as surrogate measures for fertility intentions to assess latent fertility intentions); (2) multi-source data (e.g., collecting parental reports on attachment quality to cross-verify self-reported results).

Furthermore, the sample was limited to college students in Henan Province (a moderately developed economic region in central China), which may affect generalizability. For instance, students in East China (e.g., Jiangsu) face higher living costs and may exhibit differences in fertility attitudes, while those in western regions (e.g., Sichuan) might be more influenced by traditional family values. Future studies should expand the sample scope to eastern, central, and western China, and even conduct cross-cultural comparisons (e.g., comparing Chinese and Korean college students) to verify the universality of mediating and moderating mechanisms across different regions and cultures. Additionally, the lack of significant predictive power for family strength (e.g., problem-solving skills, social support) does not indicate their practical significance, possibly due to sample characteristics (e.g., predominantly college students or young adults without post-fertility family challenges) leading to “unactivated needs.” Future research could broaden the sample to include married individuals or those with fertility plans to re-examine the role of family strength.

Finally, the “functional void” of mother attachment requires further exploration. The lack of significant predictive power in mother attachment may stem from “selection bias in mediating variables” – suggesting that its influence on fertility intentions might be mediated through factors like marital security rather than family beliefs. For instance, unmarried individuals’ fertility intentions may be more influenced by marital planning rather than family values. Future research could employ “multi-intermediary models” to comprehensively investigate the pathways through which mother attachment operates.

## 6 Conclusion

Through multivariate regression and mediation/modification analysis, this study clarifies the core path of “father attachment → family beliefs → fertility intention,” which is significantly moderated by gender. Men are more likely to develop family beliefs through father attachment, and these beliefs directly drive their fertility intentions. In contrast, women’s fertility intentions require practical considerations of childbirth costs to be enhanced. These findings not only provide new evidence for the theoretical mechanism of “parent-child relationship influencing fertility intention” but also offer practical basis for developing gender-specific and dimension-specific strategies to improve fertility intentions.

## Data Availability

The original contributions presented in this study are included in this article/supplementary material, further inquiries can be directed to the corresponding author.
