# Correction: The relationship between parent-child attachment and fertility intention: the mediating role of family resilience and the moderating role of gender

**DOI:** 10.3389/fpsyg.2025.1719802

**Published:** 2025-11-03

**Authors:** Gui-Ying Li, Zhi-qi You, Guang Hui Yang

**Affiliations:** ^1^Center for Mental Health Education for College Students, Henan Polytechnic, Zhengzhou, Henan, China; ^2^Department of Social Work, Huazhong Agricultural University, Wuhan, Hubei, China; ^3^School of Education, Anyang Normal University, Anyang, Henan, China

**Keywords:** parent-child attachment, family resilience, fertility intention, father attachment, mother attachment, family beliefs, family strength, gender

The title of this article was erroneously given as: [The relationship between parent-child attachment and fertility intention: the mediating role of family resilience and the moderating role of gender moderating]. The correct title of the article is [The relationship between parent-child attachment and fertility intention: the mediating role of family resilience and the moderating role of gender].

The original version of this article has been updated.

